# Expression of oxytocin in hypothalamus and reduction of nociceptive stress following administration of Kamikihi-to in female rats

**DOI:** 10.3389/fphar.2022.961135

**Published:** 2022-08-30

**Authors:** Takashi Maruyama, Makiko Shimizu, Naofumi Ikeda, Kazuhiko Baba, Mitsuhiro Yoshimura, Yoichi Ueta

**Affiliations:** Department of Physiology, University of Occupational and Environmental Health, Kitakyushu, Japan

**Keywords:** oxytocin, nociceptive stress, formalin test, estrus cycle, Kamikihi-to, estrogen, cholecystokinin-8, hot plate test

## Abstract

Hypothalamo-neurohypophysial oxytocin (OXT) plays an essential role in reproduction and in several socio-physiological functions, including stress reduction, anxiety relief, feeding suppression, social recognition, and trust building. Recent studies suggest that the central OXT system is also involved in antinociceptive and anti-inflammatory functions. Kamikihi-to (KKT), a Japanese traditional herbal (Kampo) medicine composed of 14 herbal ingredients, is clinically prescribed for patients with psychological symptoms, including anxiety, depression, and insomnia, and it has been associated with OXT expression. We investigated the antinociceptive response and OXT expression according to sex and the effects of KKT pre administration in a rat model. We found that nociceptive responses measured *via* the hot plate and formalin tests were attenuated following the administration of KKT-enriched feed for 4 weeks. The observation of mRFP1 fluorescence in OXT-mRFP1 transgenic rats revealed that KKT-administered rats showed increased expression of OXT in the magnocellular and parvocellular paraventricular nucleus of the hypothalamus. Food intake in the KKT-pre-administered group significantly decreased after cholecystokinin (CCK)-8 administration. Our results suggest that KKT is involved in the attenuation of nociceptive stress in female rats by enhancing the expression of OXT in the hypothalamus.

## 1 Introduction

The oxytocin (OXT) is a hypothalamo-neurohypophysial peptide composed of nine amino acids (Dale, 1906). OXT is synthesized in the paraventricular- (PVN) and supraoptic nucleus (SON) of the hypothalamus and subsequently secreted from the posterior pituitary (PP) into the systemic circulation (Sofroniew, 1980). OXT in vesicles in axon terminals is exocytosed into the blood from the PP and delivered to target organs *via* the bloodstream (hormonal regulation). OXT peptide is exocytosed from magnocellular neurosecretory neurons cell bodies and dendrites (somatodendritic release) (Tobin et al., 2011) and parvocellular OXT neurons in the PVN that directly project to the central nervous system, such as the hindbrain nucleus of the solitary tract and spinal cord (nerve regulation) (Swanson and McKellar, 1979). OXT plays an essential role in reproduction, such as regulating uterine contractions and lactation (Russell and Leng, 1998). Many recent studies report that OXT is likewise involved in socio-physiological functions, such as stress reduction, anxiety relief, feeding suppression, social recognition, and trust building (Kosfeld et al., 2005; Oettl et al, 2016; Lawson, 2017; Tang et al., 2020).

Recent studies suggest that the hypothalamo-neurohypophysial OXT/vasopressin (AVP) system and the hypothalamo-pituitary adrenal (HPA) axis are activated by acute and chronic nociceptive stimuli and are involved in the modulation of nociceptive afferent pathways (Nishimura et al., 2020). Evidence indicates that the central OXT-ergic pathways are likewise involved in antinociceptive and anti-inflammatory functions (Uvnäs-Moberg et al., 1993; Matsuura et al., 2015). OXT-containing neurosecretory cells include magnocellular neurosecretory cells in the PVN and SON and the parvocellular divisions of the PVN. OXT from magnocellular neurosecretory neurons is projected to the PP via axon delivery and stored in the PP. The stress response stimulates the secretion of OXT into the blood circulation. OXT from parvocellular neurosecretory neurons in the PVN directly projects to the central nervous system and spinal cord, and this OXT-ergic projection may be involved in nociceptive control (Matsuura et al., 2015; Eliava et al., 2016).

Kamikihi-to (KKT), a Japanese traditional herbal medicine (Kampo) composed of 14 herbal constituents, is clinically prescribed for patients with psychological symptoms, including anxiety, depression, and insomnia (Lee et al., 2018). KKT has been approved by the Ministry of Health, Labour and Welfare in Japan since 1986, with indications for anemia, insomnia, anxiety, and neurosis. Maejima et al. (2021) reported that report a relationship between KKT treatment and OXT neuron activity by electrophysiological experiments. OXT neuron in the PVN of the hypothalamus are stimulated by KKT administration, which exerts an anti-stress response and increases OXT secretion in the cerebrospinal fluid (Tsukada et al., 2021).

Some studies suggest the relationship between OXT and estrogen (Acevedo-Rodriguez et al., 2015). In females, the estrus cycle affects estrogen secretion; therefore, OXT secretion needs to be considered with respect to the effects of the estrus cycle. Since the expression of oxytocin is also affected by estrogen, one of the most important goals of this study is to evaluate the effects of KKT by sex.

In the present study, we investigated the antinociceptive response due to sex and the effects of KKT pre administration in a rat model by observing the nociceptive stress response. To validate the hypothesis that KKT promotes OXT expression in hypothalamus and attenuates nociceptive stress, we evaluated the contribution of KKT treatment to OXT neurons by observing the OXT expressions in hypothalamus after nociceptive stress. And we performed the experiment to observe the food intake after cholecystokinin (CCK)-8 intraperitoneal administration in order to evaluate reactivity of the OXT neuron in hypothalamus.

## 2 Materials and methods

### 2.1 Formalin test

The rats were unilaterally and subcutaneously injected with 2% formalin (100 μL) into the plantar surface of the left hind paw using a sterile syringe (i.e., the formalin test). Three main behavioral responses, licking, tonic flexion, and paw jerk as formalin-induced pain were mainly observed. The total amount of time that each rat spent licking was measured after watching a video recording of the behavioral observations. Formalin test procedures were performed using the same method as reported by Matsuura et al. (2015).

### 2.2 Animals

We conducted this study on adult male and female oxytocin-mRFP1 Wistar transgenic rats (weighing 250–450 g (male), 200–350 g (female)) and Wistar Imamichi rats (weighing 250–450 g (male), 200–350 g (female)). The rats were bred and housed under normal laboratory conditions (23–25°C, 12-h light/dark cycle, lights on at 0700 h) with free access to food and tap water. All rats were treated after 10 weeks of age (i.e., once the estrus cycle was established in the female rats). Female Wistar Imamichi rats that have a regular 4-days estrus cycle, were adjusted to the same phase of estrus cycle. All female rat experiments were performed in estrus period with the estrus cycle. One week prior to the day of the experiment, the estrus cycle was confirmed with a vaginal smear. And the experiment was performed after confirming the subject rat was in estrus period on the day of the experiment. This study was conducted in accordance with the guidelines on the use and care of laboratory animals established by the Physiological Society of Japan, adhering to national and international laws and guidelines for humane animal care and research ethics, and was approved by the Ethics Committee of Animal Care and Experimentation at the University of Occupational and Environmental Health in Japan. Oxytocin-mRFP1 transgenic rats were screened via polymerase chain reaction using genomic DNA extracted from ear biopsies, as described previously (Katoh et al., 2011).

### 2.3 Kamikihi-to administration

The dry bulk powdered extracts of KKT (Lot No. 381178000) used in the present study were supplied by Tsumura & Co. (Tokyo, Japan). The following 14 raw materials were used to produce the KKT powder, the formula to produce 5 g of dried extract of KKT comprised: *Astragali Radix* (3 g, root of *Astragalus propinquus* Schischkin or *Astragalus* mongholicus Bunge), *Bupleuri Radix* (3 g, root of *Bupleurum falcatum* L.), *Ziziphi Semen* (3 g, seed of *Ziziphus jujuba* var. spinosa [Bunge] Hu ex H.F.Chow), *Atractylodis Lanceae Rhizoma* (3 g, rhizome of *Atractylodes lancea* [Thunb.] DC. or *Atractylodes chinensis* [Bunge] Koidz.), *Ginseng Radix* (3 g, root of *Panax ginseng* C.A.Mey.), *Poria* (3 g, sclerotium of Wolfiporia cocos Ryvarden et Gilbertson), *Longan Arillus* (3 g, arillus of *Dimocarpus longan* Lour.), *Polygalae Radix* (2 g, root of *Polygala tenuifolia* Willd.), *Gardeniae Fructus* (2 g, fruit of *Gardenia jasminoides* J. Ellis), *Ziziphi Fructus* (2 g, fruit of *Ziziphus jujuba* var. inermis [Bunge] Rehder), *Angelicae Radix* (2 g, root of *Angelica acutiloba* [Siebold & Zucc.] Kitag. or *Angelica acutiloba* var. sugiyamae Hikino), *Glycyrrhizae Radix* (1 g, root of *Glycyrrhiza* uralensis Fisch. ex DC. or *Glycyrrhiza* glabra L.), *Zingiberis Rhizoma* (1 g, rhizome of *Zingiber officinale* Roscoe), and *Saussureae Radix* (1 g, root of Aucklandia costus Falc.). The herbs were mixed following combined extraction in boiling water. The soluble extract was separated from the insoluble waste and concentrated by removing water under reduced pressure. The concentrated extract was then spray-dried to produce the KKT powder. The raw materials were authenticated via identification of external morphology and marker compounds for plant specimens, according to the methods of the Japanese Pharmacopeia and company standards. These raw materials and production methods are same as previous study (Oizumi et al., 2020). Extract quality was standardized based on good manufacturing practices, as defined by the Ministry of Health, Labour and Welfare of Japan. The KKT was mixed with a solid-shaped feed for rodents at a concentration of 3%. The control feed was solid-shaped with the same hardness and odor as the MF feed (KBT Oriental Co. Ltd., Tosu, Japan). It has been shown that food intake and weight gain of those taking MF feed during the administration period were not different from those under the control feed. These concentrations were determined by referring to the effective dosages for KKT in our preliminary experiment.

### 2.4 Evaluation of the nociceptive threshold

The mechanical nociceptive threshold was evaluated using the manual von Frey test and the hot plate test at 52.5°C. Calibrated von Frey filaments (North Coast Medical, Gilroy, CA, United States) were used for the von Frey test, with repeated measurements performed on the same animal as per the method reported by Shir and Seltzer (1990). Mechanical stimulation of the plantar surface of the ipsilateral foot was performed using filaments ranging from 0.25 to 20.0 g. For the hot plate test, a skilled personnel manually measured latency to pain-reflex behavior in rats after placement on a heated metal plate (52.5°C). The measurements were performed twice, and the average was considered as the latency. Nociceptive threshold measurements were performed using the same method reported by Nishimura et al. (2020).

### 2.5 Measurement of plasma adrenocorticotropic hormone and corticosterone concentrations

We collected the trunk blood of rats decapitated, 30 min after formalin injection. Plasma samples were obtained by centrifugation and measured in duplicate. Plasma concentrations of adrenocorticotropic hormone (ACTH) and corticosterone (CORT) were measured using an enzyme-linked immunosorbent assay kit (Corticosterone ELISA Kit, Cayman Chem., Ann Arbor, MI, United States). The results were averaged for each group at each evaluation time point (Nishimura et al., 2020).

### 2.6 mRFP1 fluorescence measurement

The rats were deeply anesthetized via intraperitoneal administration of a combination of anesthetics. The rats were then perfused transcardially with 0.1-M phosphate buffer (PB; pH 7.4) containing heparin (1,000 U/L), followed by 4% paraformaldehyde (PFA) in 0.1-M PB. The brains were carefully removed and post-fixed with 4% PFA in 0.1-M PB for 48 h at 4°C. The tissues were then cryoprotected in 20% sucrose in 0.1-M PB for 48 h at 4°C. Following this, the tissues were cut into 30-μm sections with a microtome (Komatsu Electronics Co., Ltd., Hiratsuka, Japan). About 24 brain slices respectively which contains PVN and SON in hypothalamus were collected from each brain sample. And one sections was selected respectively for PVN and SON as the same level of each brain. The sections were observed via fluorescence microscopy (ECLIPSEE 600; Nikon Corp., Tokyo, Japan) with an RFP filter excitation/emission wavelength; 542/620 nm (Nikon Corp.), and the intensity of fluorescence in the PVN, SON, and PP was measured. The images were captured with a digital camera. We used an imaging analysis system (NIS-Elements; Nikon Corporation, Tokyo, Japan) to average the mRFP1 fluorescence intensity per unit area in the SON, magnocellular paraventricular nucleus (mPVN), and parvocellular paraventricular nucleus (pPVN) for each section.

### 2.7 c-Fos immunohistochemistry

The brain sections were incubated for 3 days at 4°C with a rabbit polyclonal c-Fos antibody (sc-52; Santa Cruz Biotechnology, Dallas, TX, United States; 1:1,000) in Phosphate-buffered saline (PBS) containing 0.3% Triton X-100 (PBST). After washing three times in 0.1-M PBS for 30 min in total, the sections were incubated overnight at 4°C with a secondary antibody (goat Alexa Fluor 488-conjugated anti-rabbit IgG antibody; Molecular Probes, Eugene, OR, United States; 1:1,000) in PBST. The sections were washed three times in 0.1-M PBS for 30 min in total. The sections were then observed via fluorescence microscopy (ECLIPSEE 600; Nikon Corp., Tokyo, Japan) with a GFP filter (Nikon Corp.). cFos-immunoreactive cells in the PVN and SON were counted for each captured image, as described previously (Motojima et al., 2016).

### 2.8 Statistical analysis

All data are reported as mean ± standard error of the mean. The reported value is averaged across animals in each group. Statistical significance was analyzed using one-way analysis of variance to examine changes over the course of the experiment, using Tukey-Kramer-type adjustment for multiple comparisons (JMP 11.0.0; SAS Institute, Inc., Cary, NC, United States). *p*-values of < 0.05 were considered statistically significant.

## 3 Results

### 3.1 Kamikihi-to administration and the mechanical nociceptive threshold

**FIGURE 1:**
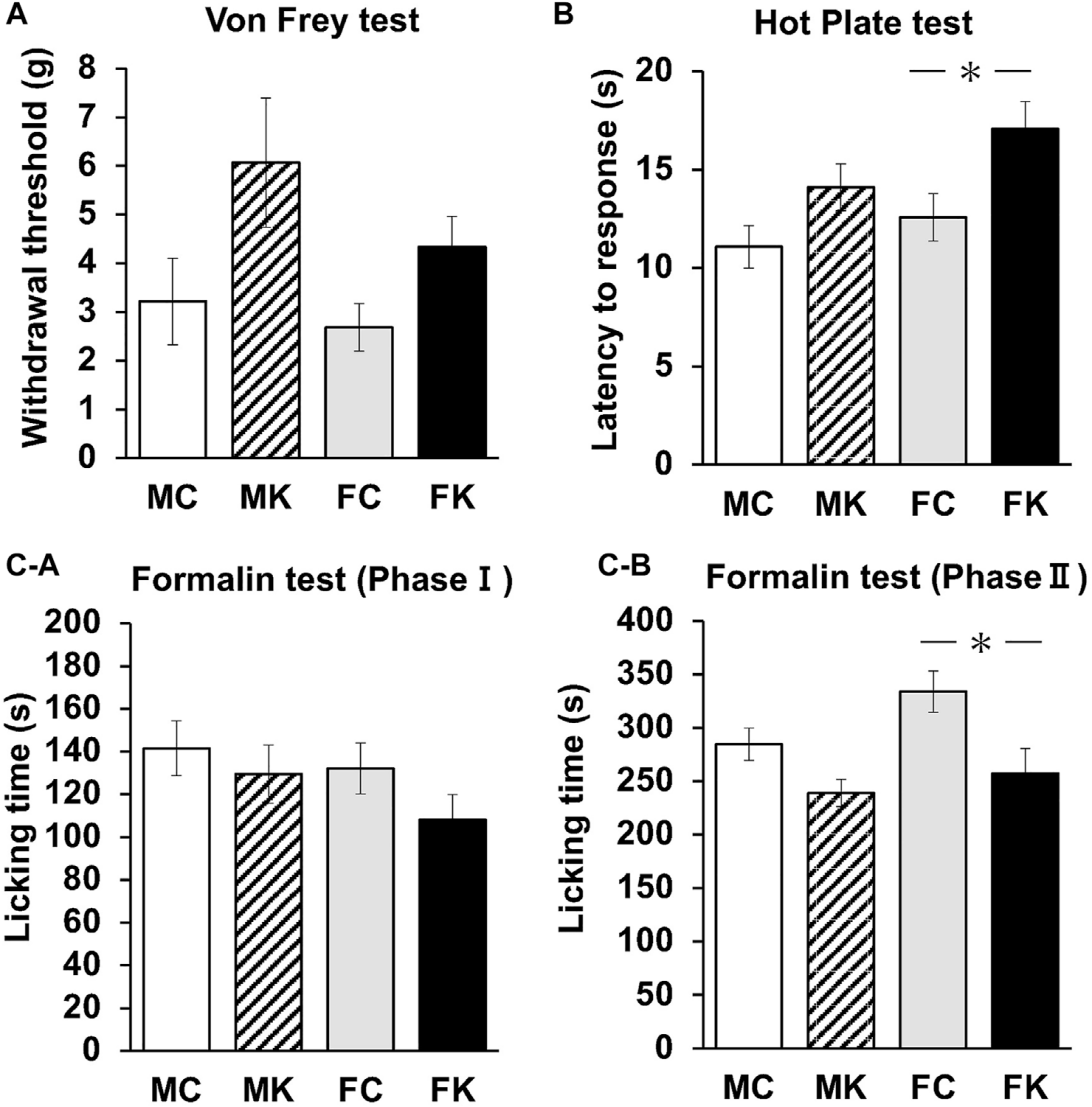
Changes in the mechanical nociceptive threshold as measured by von Frey **(A)** and hot plate **(B)** tests of the male control group (MC), male Kamikihi-to (KKT)-treated group (MK), female control group (FC) and female KKT-treated group (FK) (*n* = 12 per group at each test). The time course of the development of the formalin response (i.e., licking time) was measured. **(C-a)** Phase I (0–10 min after subcutaneous injection of 2% formalin) and phase II (10–40 min after subcutaneous injection of formalin) were calculated **(C-b)**. The data are presented as the mean ± standard error of the mean. *p < 0.05 compared between the control and KKT groups.

Four groups (males with control feed [MC], males with KKT administration [MK], females with control feed [FC], and females with KKT administration [FK]) were evaluated for mechanical nociceptive response with the von Frey, hot plate, and formalin tests (*n* = 12 per group for each test). The KKT group was administered a feed containing 3% KKT for 4 weeks prior to each experiment. In the von Frey test, the threshold did not change when comparing control and KKT rats or male and female rats (F [3, 44] = 4.4236, *p* = 0.0084) (Figure 1A). In the hot plate test, the latency to response significantly increased in the FK group compared with that in the FC group (F [3, 44] = 4.3802, *p* = 0.0088, FC vs. FK; *p* = 0.0129*) (Figure 1B). Measures of nociception via formalin tests showed that female rats in the KKT-treated group significantly decreased their licking time during the chronic (10–40 min) phases of the formalin test (Phase II [10–40 min after subcutaneous injection of formalin]: F [3, 44] = 5.2087, *p* = 0.0036, FC vs. FK; *p* = 0.0228) (Figures 1B,C). There were no statistically significant differences in the acute (0–10 min) phases of the formalin test in each group (Phase I [0–10 min after subcutaneous injection of 2% formalin]: F [3, 44] = 1.2542, *p* = 0.3017) (Figures 1A–C).

### 3.2 Plasma adrenocorticotropic hormone and corticosterone after formalin injection

**FIGURE 2:**
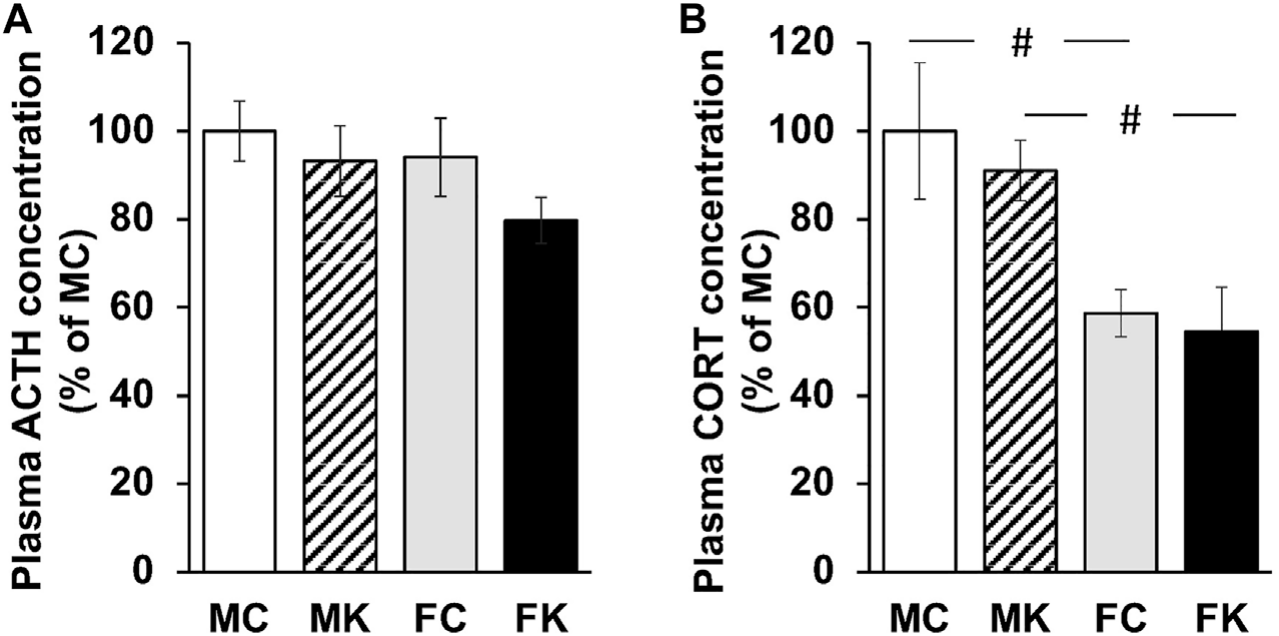
Plasma ACTH concentration **(A)** and Plasma CORT concentration **(B)** 30 min after subcutaneous injection of 2% formalin. The data are presented as the mean ± standard error of the mean. #*p* < 0.05 compared between male and female rats. ACTH, adrenocorticotropic hormone; CORT, corticosterone; MC, male control group; MK, male Kamikihi-to-treated group; FC, female control group; FK, female Kamikihi-to-treated group.

The plasma concentrations of ACTH and CORT were measured 30 min after formalin injection. The plasma concentrations of ACTH did not significantly decrease in any group (F [3, 19] = 1.3176, *p* = 0.2979) (Figure 2A). There were likewise no statistically significant differences in the plasma concentrations of CORT when comparing the control and KKT groups. The plasma concentrations of CORT in female rats were significantly lower than those in male rats (F [3, 20] = 5.0171, *p* = 0.0094, MC vs. FC; *p* = 0.0095**, MK vs. FK; *p* = 0.0198*) (Figure 2B).

### 3.3 Effect of Kamikihi-to on OXT-mRFP1 fluorescence

**FIGURE 3:**
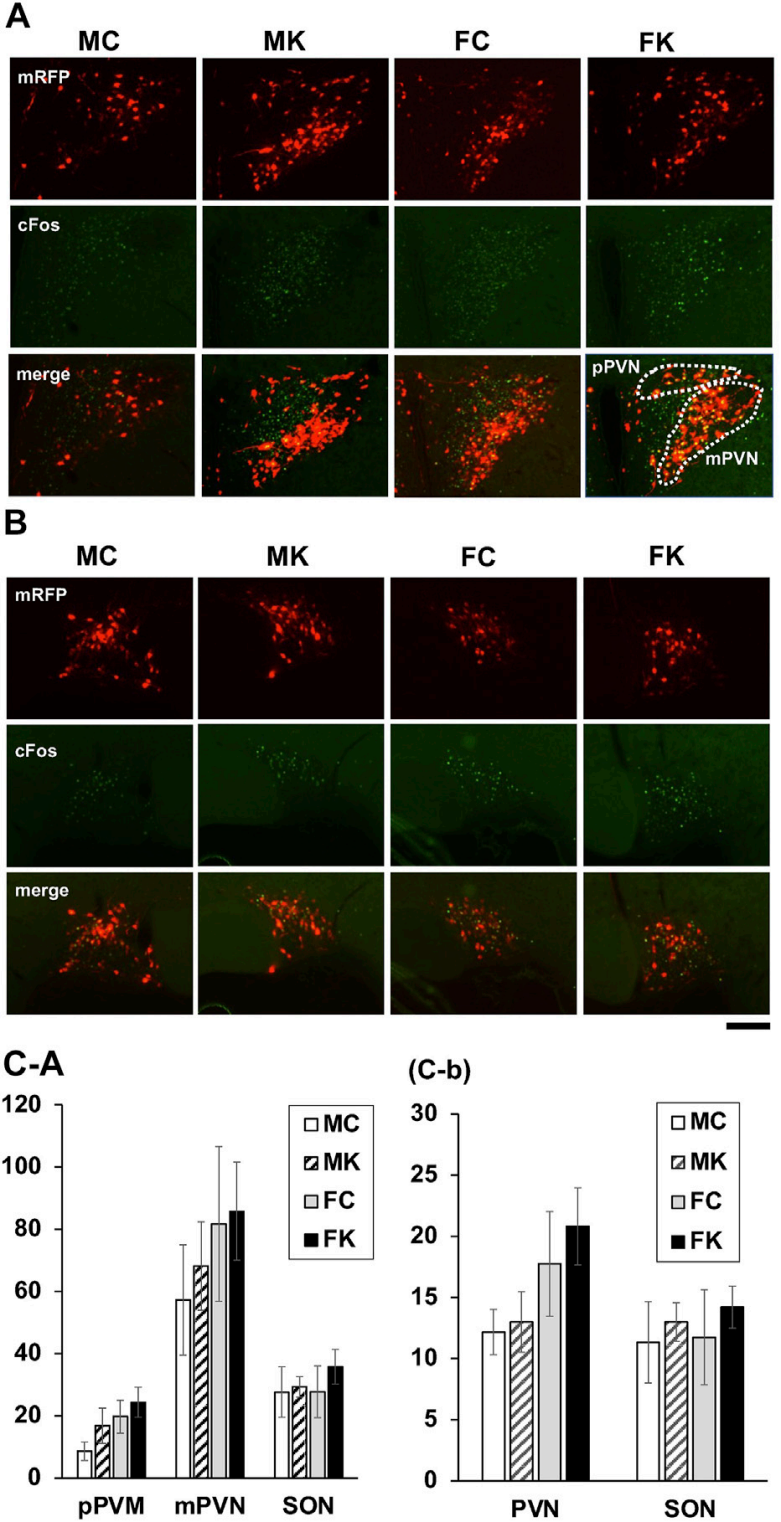
Oxytocin expression in hypothalamus **(A)** Oxytocin-monomeric red fluorescent protein 1 (OXT-mRFP1) fluorescence was observed by fluorescence microscopy in the parvocellular paraventricular nucleus (pPVN), magnocellular paraventricular nucleus (mPVN), supraoptic nucleus (SON) and Posterior Pituitary (PP) in each the male control group (MC), male Kamikihi-to (KKT)-treated group (MK), female control group (FC) and female KKT-treated group (FK) (*n* = 6 per group at each test). The images are represented in grayscale, white is high intensity of red fluorescent. Scale bars = 100 μm. **(B)** Intensity of mRFP1 fluorescence was observed and is presented as % of male control group. The data are presented as the mean ± standard error of the mean. *p < 0.05 compared to male control group.

mRFP1 fluorescence was observed via fluorescence microscopy in the pPVN, mPVN, SON, and PP in all the four groups–MC, MK, FC, and FK (*n* = 6 per group for each test; Figure 3A). mRFP1 fluorescence was significantly higher in the FK group than in the FC group in the pPVN (F [3, 20] = 3.3849, *p* = 0.0383, FC vs. FK; *p* = 0.0280*). mRFP1 fluorescence in the MK group was also significantly higher than in the MC group, and the fluorescence in the FK group was significantly higher than in the FC group in the mPVN (F [3, 20] = 4.2062, *p* = 0.0185, MC vs. MK; *p* = 0.0471*, FC vs. FK; *p* = 0.0114*) (Figure 3B). In the SON and PP, the KKT-treated group exhibited higher fluorescence than the control group in both male and female rats, but the difference was not statistically significant (SON: F [3, 20] = 2.0373, *p* = 0.1410; PP: F [3, 20] = 2.8458, *p* = 0.0635] (Figure 3B).

### 3.4 Effects of Cholecystokinin (CCK) -8 administration with Kamikihi-to

**FIGURE 4:**
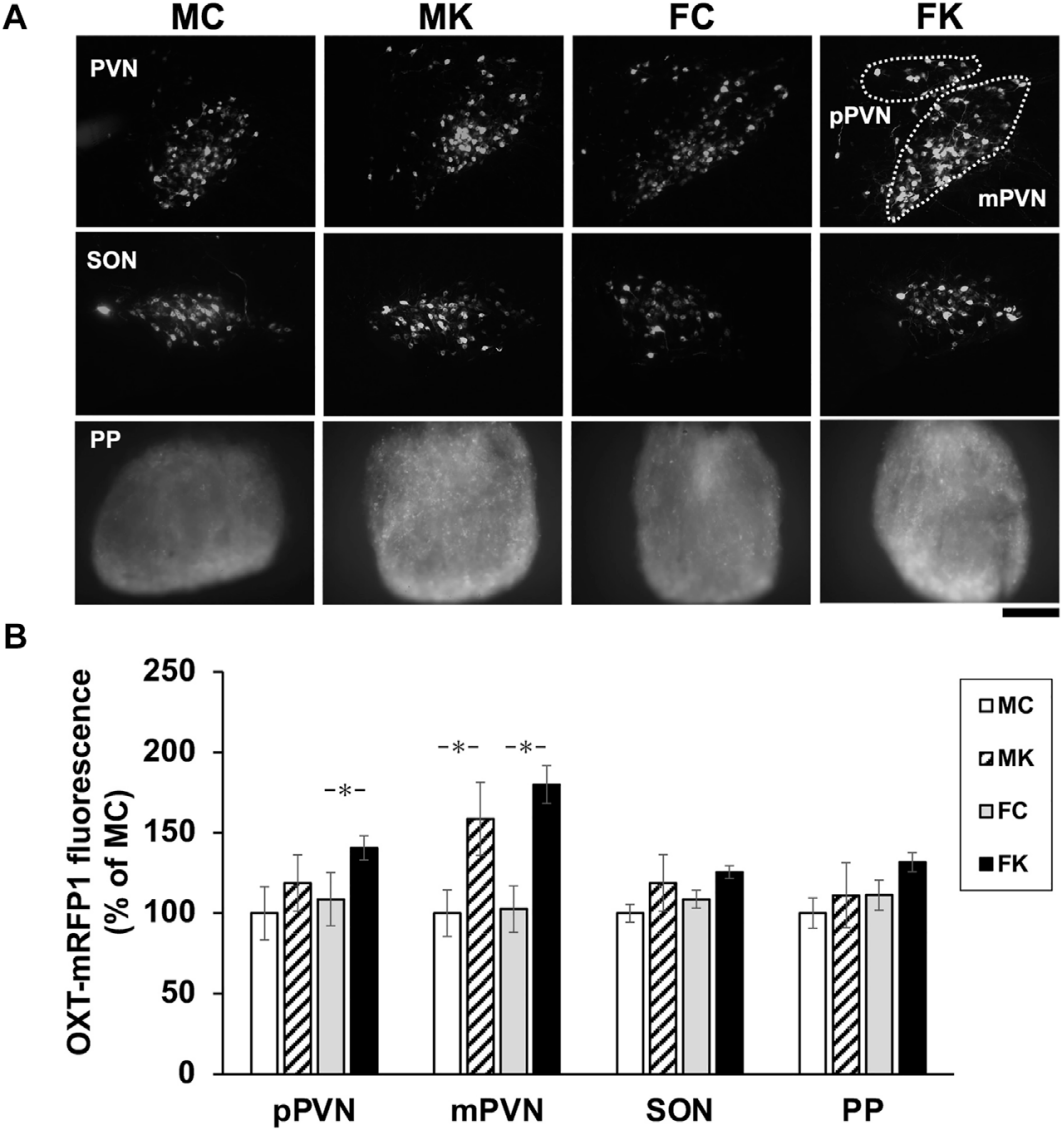
Time-course studies showing the effects of intraperitoneal administration of saline or cholecystokinin (CCK)-8 (20 g/kg) on cumulative food intake in the control group and Kamikihi-to (KKT)-treated group on male rat **(A)** and female rat **(B)**. Data for cumulative food and are presented as the mean ± standard error of the mean (each group, *n* = 6). *p < 0.05 compared between the control and KKT groups; ^#^ *p* < 0.05 compared between the saline and CCK groups.

Intraperitoneal saline (vehicle) or cholecystokinin (CCK)-8 (Peptide Institute, Osaka, Japan) for each group (control and 3% KKT-treated rats) was administered, and food intake was measured at 0, 0.5, 1.0, 1.5, 3.0, and 6.0 h, respectively, following its administration. This experiment was performed on male rats (*n* = 6 for each group) and female rats (*n* = 6 for each group) during the estrus period on the day of the experiment. For the experiment of female rat, Cumulative food intake was significantly decreased 1.0, 1.5, and 3.0 h after intraperitoneal administration for the CCK-8-treated group (1.0 h: F [3, 20] = 5.7778, *p* = 0.0052; 1.5 h: F [3, 20] = 5.4961, *p* = 0.0064; 3.0 h: F [3, 20] = 2.9865, *p* = 0.0556). With respect to the effects of KKT administration, there was a statistically significant decrease in food intake at 1.0 h following intraperitoneal CCK-8 administration in the KKT group compared to that in the control group (control/CCK vs. KKT/CCK; *p* = 0.0389*) (Figure 4B). On the other hand, no significant difference was observed in the KKT group compared to that in the control group in male rats (Figure4A).

**FIGURE 5:**
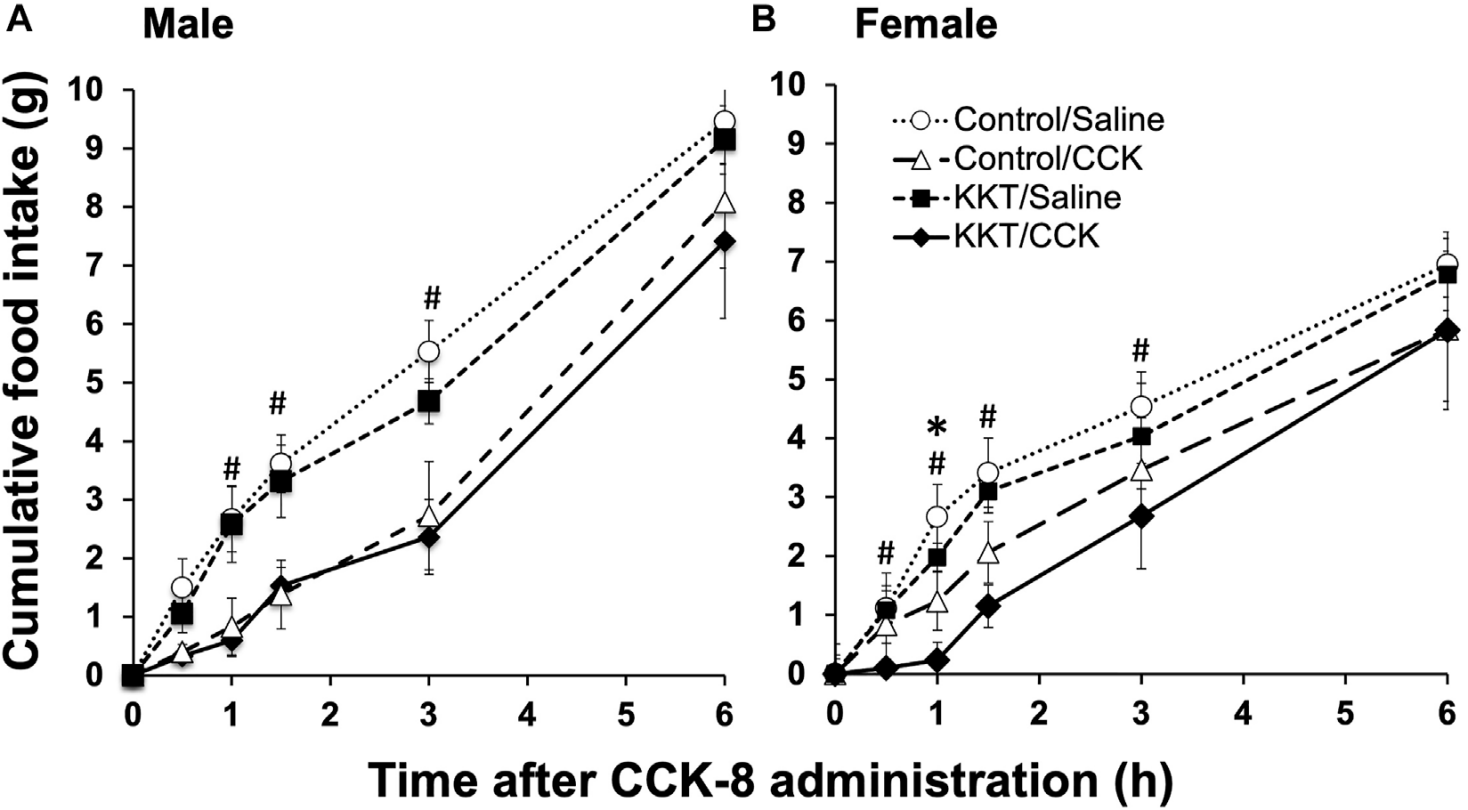
Endogenous oxytocin-monomeric red fluorescent protein 1 (OXT-mRFP1; red), Fos-immunoreactive (-IR) cells (green) and the merged image of the paraventricular nucleus **(A)** and supraoptic nucleus (SON) **(B)**. Sections were obtained 90 min after intraperitoneal cholecystokinin-8 injection for the male control group (MC), male KKT-treated group (MK), female control group (FC), and female KKT-treated group (FK) (MC: *n* = 6, MK: *n* = 6, FC: *n* = 4, FK: *n* = 5). Fos IR cell were counted in the parvocellular paraventricular nucleus (pPVN), magnocellular paraventricular nucleus (mPVN), and the SON region **(C-a)**. Cells expressing both nuclear Fos-IR and cytoplasmic OXT-mRFP1 were counted **(C-b)**.

Immunohistochemistry for c-Fos was observed in the brain sections sampled 90 min after intraperitoneal administration of CCK-8 in male and female mRFP1 transgenic rats. The number of c-Fos-positive neurons in the pPVN, mPVN, and SON in the control group and in the KKT-treated group following 4 weeks of KKT administration was counted (Figure 5A, B. The average count of c-Fos-positive neurons in the KKT group was higher than that in the control group and the same count in male rats was higher than that in female rats, but there were no statistically significant differences in the average c-Fos-positive neuron counts in the mPVN, pPVN, and SON when comparing the control and KKT groups (among both male and female rats) (mPVN: F [3, 17] = 2.0110, *p* = 0.1506; pPVN: F [3, 17] = 0.5500, *p* = 0.6549; SON: F [3, 17] = 0.3379, *p* = 0.7982) (Figure 5C-a). The number of c-Fos-positive neurons that merged with OXT-mRFP1 neurons was also counted, and there were no statistically significant differences observed between the groups (Figure 5C-b).

## 4 Discussion

In the nociceptive evaluations performed in this study, the latency to response in the hot plate test significantly increased in the KKT-administrated female group (FK) compared to that in the female control group (FC), and licking time in the formalin test (Phase II) was significantly decreased in the FK group compared to that in the FC group (Figures 1B,C-b). These results indicate that the nociceptive response was attenuated by KKT administration. This may be an effect of spinal pain modulation and stress reduction through the HPA axis. Formalin-induced activation of the endogenous hypothalamo-neurohypophysial system caused an elevation in plasma OXT levels and that the OXT-ergic spinal pathway, and the OXT-ergic activation were involved in pain modulation via oxytocin receptor in the spinal cord (Matsuura et al., 2016).

Notably, an attenuation of the nociceptive response and enhancement of OXT neurons after KKT treatment was observed in female rats in this study. Many previous clinical and experimental studies have shown that traditional Japanese herbal medicine (Kampo) is particularly effective for women with symptoms such as those caused by menopause. Kampo may play an auxiliary role in the action of estrogen (Ushiroyama, 2013; Johnson et al., 2019). KKT has been found to be effective for the treatment of behavioral and circulatory disorders in an ovariectomized rat model (Shimamura et al., 1996). Oxytocin expression can be affected by estrogen since estrogen receptors are located on OXT neurons in the PVN (Sloan et al., 2018). Rats subjected to ovariectomy show a significantly decreased OXT expression in the hypothalamus (De Melo et al., 2016); therefore, OXT expression may be regulated by estrogen and OXT expression is thought to be higher during the estrus period. AVP is a hypothalamo-neurohypophysial peptide very similar to oxytocin, Nishimura et al. reported that sex-based differences in the dynamics of hypothalamo-neurohypophysial AVP, which indicate that hypothalamo-neurohypophysial peptides can be affected by estrogen-dependent regulation (Nishimura et al., 2019). Hypothalamo-neurohypophysial OXT and AVP may have estrogen-dependent regulation; therefore, the larger effect on female rats in this experiment suggests that KKT enhanced the action of OXT neurons via estrogen and showed a pain-attenuating effect.

The sex-based differences should be considered while evaluating nociceptive response as well, as in OXT expression in the present study. Regarding sex-based differences in the nociceptive response, it was reported that the pain threshold in female rats depends on the estrus state (Ibironke and Aji, 2011). Licking time in the formalin test significantly decreased from the metestrus to the diestrus phase compared with that in the proestrus and estrus phases, which was due to an increased pain threshold (connoting a decrease in pain sensitivity) (Ibironke and Aji, 2011). In the present study, the estrus state of female rats on the experiment day was adjusted to the estrus phase. A previous study indicated that nociceptive sensitivity may be higher in the estrus phase than in other estrus states; however, the differences in nociceptive responses between male and female rats were not statistically significant. There was a significant difference in plasma CORT concentrations between males and females in both the control and KKT pre-treated groups (Figure 2).

Tsukada et al. (2021) reported that ACTH and CORT levels of KKT-administrated rats slightly increased in the acute immobilization stress (AIS) group compared with those in the control group. This is a different result of the nociceptive response than in the present experiment. Our study showed no significant difference between the control and KKT groups, and there was a significant decrease in CORT levels in female rats. Oxytocin is reported to reduce stress levels via the regulation of HPA axis activity. Treatment with exogenous oxytocin reduced the level of stress-induced ACTH and the subsequent release of CORT (Windle et al., 1997; Parker et al., 2005). Oxytocin receptor blockade in the brain activates the HPA axis and the release of ACTH and CORT into the blood (Neumann et al., 1999). The reduction in HPA axis reactivity by oxytocin may be a consequence of the oxytocinergic suppression of their upstream corticotropin-releasing factor neuron activity (Pati et al., 2020). It is suggested that KKT attenuates nociceptive stress response via the central nervous system. Several studies have reported that KKT improves brain function and action in the central nervous system. KKT influences memory enhancement (Watari et al., 2015) and ameliorates the impairment of spatial memory. Moreover, the possibility of using KKT to treat memory deficits has been suggested (Egashira et al., 2007). The central mechanisms of KKT require further study.

In the present study, mRFP1 fluorescence evaluation revealed that KKT administration for 4 weeks increased the expression of OXT in both the magnocellular and parvocellular PVN divisions (Figure 3). By Using OXT-mRFP1 rats, we can identify the OXT neuron easily and see changes in the neuron’s activity and release of OXT (Hashimoto et al., 2014). While KKT administration alone does not attenuate food intake though OXT neurons are activated by KKT. Maejima et al. reported that oxytocin had a weight-loss effect only in high-fat diet (Maejima et al., 2017). It is considered that there is any mechanism in which the feeding suppression function of oxytocin works only when there is some kind of stimulus. It was found that the OXT expression increased by CCK-8 intra-peritoneal administration mainly enhanced both the magnocellular and parvocellular PVN regions. The gastrointestinal hormone, CCK-8, acts on OXT neurons in the hypothalamus via the NTS to increase blood OXT concentrations and suppress appetite (Ebenezer, 1996). We demonstrated that food consumption in the KKT-treated group was lower than that in the control group 1.0 h after CCK-8 administration. Thus, it indicates that this reduction in feed intake was induced by OXT neuron activation after CCK-8 administration. These results suggest that the reactivity of OXT neurons may be enhanced by KKT administration. These findings also indicate that KKT administration improved the responsiveness of OXT neurons in both the magnocellular and parvocellular regions of the PVN, leading to nociceptive reduction. Administration of KKT reduced acute immobilization stress (AIS)-induced defecation, and the effect of KKT was inhibited by the administration of an oxytocin receptor antagonist. Therefore, it is suggested that KKT exhibits anti-stress response and leads to increased oxytocin secretion (Lee et al., 2018). This experiment has not revealed how KKT acts on the hypothalamus in CNS. There are no previous studies which have directly proven that KKT passes through the BBB. Maejima et al. reported that KKT can directly activate PVN Oxt neurons by interacting with OTR. KKT has seven chemical components (rutin, ursolic acid, (Z)-butylidenephtalide, p-cymene, senkunolide, [6]-shogaol, [8]-shogaol) of three ingredients (Zizyphi Fructus, Angelicae Acutilobae Radix, Zingiberis Rhizoma). As for each component, some substances have been reported to be transferred to BBB. Quercetin: a metabolite of lutin (Ishisaka et al., 2011), senkyunolide A (Zuo et al., 2012), [6]-shogaol, [8]-shogaol (Simon et al., 2020).

In the present study, there were no significant differences in the nociceptive response and OXT expression observed by mRFP1 fluorescence, which was an indicator of OXT expression (Figure 3). Decreased CORT levels suggest a mechanism wherein stress response may be reduced by estrogen during the estrus period. It is also suggesting that estrogen may suppress stress hormones via OXT expression. Further studies are needed to identify sex-based differences and estrogen dependence in OXT expression. In conclusion, the present study supports the hypothesis that KKT is involved in reducing the pain response in female rats by increasing the expression of OXT in the hypothalamus.

The present study is limited by a few factors such as the lack of continuous measurement of the HPA, CORT, and OXT levels in blood or cerebrospinal fluid; the lack of an antagonist control that blocks the action of OXT; a lack of statistically sufficient number of animals to evaluate the stress response; a lack of cellular signaling analysis; and the use of rats as a model organism. Therefore, further studies in rat model are necessary and in a human model are warranted to confirm our findings and validate the effects observed. Future studies in this regard are eagerly awaited.

## Data Availability

The original contributions presented in the study are included in the article/supplementary materials, further inquiries can be directed to the corresponding author.

## References

[B1] Acevedo-Rodriguez A. Mani S. K. Handa R. J. (2015). Oxytocin and estrogen receptor β in the brain: An overview. Front. Endocrinol. 6, 160. 10.3389/fendo.2015.00160 PMC4606117 26528239

[B2] Dale H. H. (1906). On some physiological actions of ergot. J. Physiol. 31 (3), 163–206. 10.1113/jphysiol.1906.sp001148 PMC1465771 16992821

[B3] De Melo V. U. Saldanha R. R. Dos Santos C. R. De Campos Cruz J. Lira V. A. Santana-Filho V. J. (2016). Ovarian hormone deprivation reduces oxytocin expression in paraventricular nucleus preautonomic neurons and correlates with baroreflex impairment in rats. Front. Physiol. 7, 461. 10.3389/fphys.2016.00461 27790154 PMC5063006

[B4] Ebenezer I. S. (1996). Systemic administration of cholecystokinin (CCK) inhibits operant water intake in rats: Implications for the CCK-satiety hypothesis. Proc. Biol. Sci. 263, 491–496. 10.1098/rspb.1996.0074 8637930

[B5] Egashira N. Manome N. Kurauchi K. Matsumoto Y. Iwasaki K. Mishima K. (2007). Kamikihi-to, a Kampo medicine, Ameliorates impairment of spatial memory in rats. Phytother. Res. 21, 126–129. 10.1002/ptr.2034 17117451

[B6] Eliava M. Melchior M. Knobloch-Bollmann H. S. Wahis J. da Silva Gouveia M. Tang Y. (2016). A new population of parvocellular oxytocin neurons controlling magnocellular neuron activity and inflammatory pain processing. Neuron 89 (6), 1291–1304. 10.1016/j.neuron.2016.01.041 26948889 PMC5679079

[B7] Hashimoto H. Matsuura T. Ueta Y. (2014). Fluorescent visualization of oxytocin in the hypothalamo-neurohypophysial system. Front. Neurosci. 23 (8), 213. 10.3389/fnins.2014.00213 PMC4107947 25100939

[B8] Ibironke G. F. Aji K. E. (2011). Pain threshold variations in female rats as a function of the estrus cycle. Niger. J. Physiol. Sci. 26, 67–70. 22314990

[B9] Ishisaka A. Ichikawa S. Sakakibara H. Piskula M. K. Nakamura T. Kato Y. (2011). Accumulation of orally administered quercetin in brain tissue and its antioxidative effects in rats. Free Radic. Biol. Med. 51 (7), 1329–1336. 10.1016/j.freeradbiomed.2011.06.017 21741473

[B10] Johnson A. Roberts L. Elkins G. (2019). Complementary and alternative medicine for menopause. J. Evid. Based. Integr. Med. 24, 2515690X19829380. 10.1177/2515690X19829380 PMC6419242 30868921

[B11] Katoh A. Fujihara H. Ohbuchi T. Onaka T. Hashimoto T. Kawata M. (2011). Highly visible expression of an oxytocin-monomeric red fluorescent protein 1 fusion gene in the hypothalamus and posterior pituitary of transgenic rats. Endocrinology 152, 2768–2774. 10.1210/en.2011-0006 21540286

[B12] Kosfeld M. Heinrichs M. Zak P. J. Fischbacher U. Fehr E. (2005). Oxytocin increases trust in humans. Nature 435, 673–676. 10.1038/nature03701 15931222

[B13] Lawson E. A. (2017). The effects of oxytocin on eating behaviour and metabolism in humans. Nat. Rev. Endocrinol. 13 (12), 700–709. 10.1038/nrendo.2017.115 28960210 PMC5868755

[B14] Lee J. Y. Oh H. K. Ryu H. S. Yoon S. S. Eo W. Yoon S. W. (2018). Efficacy and safety of the traditional herbal medicine, gamiguibi-tang, in patients with cancer-related sleep disturbance: A prospective, randomized, wait-list-controlled, pilot study. Integr. Cancer Ther. 17, 524–530. 10.1177/1534735417734914 29034740 PMC6041922

[B15] Maejima Y. Aoyama M. Sakamoto K. Jojima T. Aso Y. Takasu K. (2017). Impact of sex, fat distribution and initial body weight on oxytocin's body weight regulation. Sci. Rep. 7 (1), 8599. 10.1038/s41598-017-09318-7 28819236 PMC5561196

[B16] Maejima Y. Horita S. Yokota S. Ono T. Proks P. Yoshida-Komiya H. (2021). Identification of oxytocin receptor activating chemical components from traditional Japanese medicines. J. Food Drug Anal. 29, 653–675. 10.38212/2224-6614.3381 35649140 PMC9931015

[B17] Matsuura T. Kawasaki M. Hashimoto H. Ishikura T. Yoshimura M. Ohkubo J.-I. (2015). Fluorescent visualisation of oxytocin in the hypothalamo-neurohypophysial/-spinal pathways after chronic inflammation in oxytocin-monomeric red fluorescent protein 1 transgenic rats. J. Neuroendocrinol. 27, 636–646. 10.1111/jne.12290 25943916

[B18] Matsuura T. Kawasaki M. Hashimoto H. Yoshimura M. Motojima Y. Saito R. (2016). Possible involvement of the rat hypothalamo-neurohypophysial/-spinal oxytocinergic pathways in acute nociceptive responses. J. Neuroendocrinol. 28, 12396. 10.1111/jne.12396 27144381

[B19] Motojima Y. Kawasaki M. Matsuura T. Saito R. Yoshimura M. Hashimoto H. (2016). Effects of peripherally administered cholecystokinin-8 and secretin on feeding/drinking and oxytocin-mRFP1 fluorescence in transgenic rats. Neurosci. Res. 109, 63–69. 10.1016/j.neures.2016.02.005 26919961

[B20] Neumann I. D. Torner L. Wigger A. (1999). Brain oxytocin: Differential inhibition of neuroendocrine stress responses and anxiety-related behaviour in virgin, pregnant and lactating rats. Neuroscience 95, 567–575. 10.1016/S0306-4522(99)00433-9 10658637

[B21] Nishimura H. Kawasaki M. Matsuura T. Suzuki H. Motojima Y. Baba K. (2020). Acute mono-arthritis activates the neurohypophysial system and hypothalamo-pituitary adrenal axis in rats. Front. Endocrinol. 11, 43. 10.3389/fendo.2020.00043 PMC7026388 32117068

[B22] Nishimura K. Yoshino K. Sanada K. Beppu H. Akiyama Y. Nishimura H. (2019). Effect of oestrogen-dependent vasopressin on HPA axis in the median eminence of female rats. Sci. Rep. 9, 5153. 10.1038/s41598-019-41714-z 30914732 PMC6435644

[B23] Oettl L. L. Ravi N. Schneider M. Scheller M. F. Schneider P. Mitre M. (2016). Oxytocin enhances social recognition by modulating cortical control of early olfactory processing. Neuron 90 (3), 609–621. 10.1016/j.neuron.2016.03.033 27112498 PMC4860033

[B24] Oizumi H. Miyazaki S. Tabuchi M. Endo T. Omiya Y. Mizoguchi K. (2020). Kamikihito enhances cognitive functions and reward-related behaviors of aged C57bl/6J mice in an automated behavioral assay system. Front. Pharmacol. 11, 1037. 10.3389/fphar.2020.01037 32765263 PMC7379479

[B25] Parker K. J. Buckmaster C. L. Schatzberg A. F. Lyons D. M. (2005). Intranasal oxytocin administration attenuates the ACTH stress response in monkeys. Psychoneuroendocrinology 30, 924–929. 10.1016/j.psyneuen.2005.04.002 15946803

[B26] Pati D. Harden S. W. Sheng W. Kelly K. B. de Kloet A. D. Krause E. G. (2020). Endogenous oxytocin inhibits hypothalamic corticotrophin-releasing hormone neurones following acute hypernatraemia. J. Neuroendocrinol. 32 (3), e12839. 10.1111/jne.12839 32133707 PMC7384450

[B27] Russell J. Leng G. (1998). Sex, parturition and motherhood without oxytocin? J. Endocrinol. 157, 343–359. 10.1677/joe.0.1570343 9691969

[B28] Shimamura M. Nishizawa K. Yamashita A. (1996). Effects of Kamikihi-to on ovariectomy-induced changes in behavior and circulation in rats. Nihon Yakurigaku Zasshi. 108, 65–75. 10.1254/fpj.108.65 8827724

[B29] Shir Y. Seltzer Z. (1990). A-fibers mediate mechanical hyperesthesia and allodynia and C-fibers mediate thermal hyperalgesia in a new model of causalgiform pain disorders in rats. Neurosci. Lett. 115, 62–67. 10.1016/0304-3940(90)90518-E 2216058

[B30] Simon A. Darcsi A. Kéry Á. Riethmüller E. (2020). Blood-brain barrier permeability study of ginger constituents. J. Pharm. Biomed. Anal. 177, 112820. 10.1016/j.jpba.2019.112820 31476432

[B31] Sloan D. K. Spencer D. S. Curtis K. S. (2018). Estrogen effects on oxytocinergic pathways that regulate food intake. Horm. Behav. 105, 128–137. 10.1016/j.yhbeh.2018.08.007 30118729

[B32] Sofroniew M. V. (1980). Projections from vasopressin, oxytocin, and neurophysin neurons to neural targets in the rat and human. J. Histochem. Cytochem. 28 (5), 475–478. 10.1177/28.5.7381192 7381192

[B33] Swanson L. W. McKellar S. (1979). The distribution of oxytocin- and neurophysin-stained fibers in the spinal cord of the rat and monkey. J. Comp. Neurol. 188, 87–106. 10.1002/cne.901880108 115910

[B34] Tang Y. Benusiglio D. Lefevre A. Hilfiger L. Althammer F. Bludau A. (2020). Social touch promotes interfemale communication via activation of parvocellular oxytocin neurons. Nat. Neurosci. 23 (9), 1125–1137. 10.1038/s41593-020-0674-y 32719563

[B35] Tobin V. A. Douglas A. J. Leng G. Ludwig M. (2011). The involvement of voltage-operated calcium channels in somato-dendritic oxytocin release. PLoS One 6, e25366. 10.1371/journal.pone.0025366 22028774 PMC3197583

[B36] Tsukada M. Ikemoto H. Lee X.-P. Takaki T. Tsuchiya N. Mizuno K. (2021). Kamikihito, a traditional Japanese Kampo medicine, increases the secretion of oxytocin in rats with acute stress. J. Ethnopharmacol. 276, 114218. 10.1016/j.jep.2021.114218 34029638

[B37] Ushiroyama T. (2013). The role of traditional Japanese medicine (kampo) in the practice of psychosomatic medicine: The usefulness of kampo in the treatment of the stress-related symptoms of women, especially those with peri-menopausal disorder. Biopsychosoc. Med. 7, 16. 10.1186/1751-0759-7-16 24148283 PMC4016593

[B38] Uvnäs-Moberg K. Bruzelius G. Alster P. Lundeberg T. (1993). The antinociceptive effect of non-noxious sensory stimulation is mediated partly through oxytocinergic mechanisms. Acta Physiol. Scand. 149, 199–204. 10.1111/j.1748-1716.1993.tb09612.x 8266809

[B39] Watari H. Shigyo M. Tanabe N. Tohda M. Cho K.-H. Kyung P. S. (2015). Comparing the effects of kamikihito in Japan and kami-guibi-tang in Korea on memory enhancement: Working towards the development of a global study. Phytother. Res. 29, 351–356. 10.1002/ptr.5250 25346293

[B40] Windle R. J. Shanks N. Lightman S. L. Ingram C. D. (1997). Central oxytocin administration reduces stress-induced corticosterone release and anxiety behavior in rats. Endocrinology 138, 2829–2834. 10.1210/endo.138.7.5255 9202224

[B41] Zuo A. H. Cheng M. C. Wang L. Xiao H. B. (2012). [Analysis of chemical constituents of chuanxiong rhizoma absorbed into rat brain tissues by UPLC-Q-TOF-MS]. Zhongguo Zhong Yao Za Zhi 37 (23), 3647–3650. Chinese. PMID: 23477157. 23477157

